# 142. Effect of Discontinuation of Antimicrobial Stewardship Programs on the Antibiotic Usage Pattern and Incidence of Antibiotic Resistance in the Major Bacterial Species

**DOI:** 10.1093/ofid/ofab466.344

**Published:** 2021-12-04

**Authors:** Wooyoung Jang, Hyeonjun Hwang, Hyun-uk Jo, Yong-Han Cha, Bongyoung Kim

**Affiliations:** 1 Hanyang University College of Medicine, Seoul, Seoul-t’ukpyolsi, Republic of Korea; 2 Korea Institute for Industrial Economics and Trade, Sejong, Ch’ungch’ong-namdo, Republic of Korea; 3 Good Munhwa Hospital, Busan, Pusan-jikhalsi, Republic of Korea; 4 Eulji University Hospital, Daejeon, Taejon-jikhalsi, Republic of Korea

## Abstract

**Background:**

The aim of this study was to analyze the effect of discontinuation of antimicrobial stewardship programs (ASP) activity on the antibiotic usage pattern.

**Methods:**

An interrupted time series analysis assessing the trends in antibiotic use and incidence of antimicrobial resistance in major pathogens was conducted between March 2017 and April 2019 in an 859-bed university-affiliated hospital in Korea, where all ASP activities were discontinued in February 2018. The major activity of the ASP was a restrictive measure for designated antibiotics. We defined antibiotics as medication with the Anatomical Therapeutic Chemical class J01, and the antibiotic consumption was measured as days of therapy (DOT), which was then standardized per 1,000 patient-days.

**Results:**

The use of antibiotics against multidrug-resistant pathogens increased immediately after the discontinuation of restrictive antibiotic program (41.01 and 150.99 days of therapy [DOT]/1,000 patient-days in the general ward [GW] and intensive care unit [ICU], respectively). In addition, there were positive changes for the GW and ICU (4.20 and 31.57 DOT/1,000 patient-days per month, respectively). The use of broad-spectrum antibiotics in patients in the ICU significantly decreased (-674.26 DOT/1,000 patient-days). For non-broad-spectrum antibiotics, there were positive changes for the GW and ICU (18.17 and 22.69 DOT/1,000 patient-days per month, respectively.

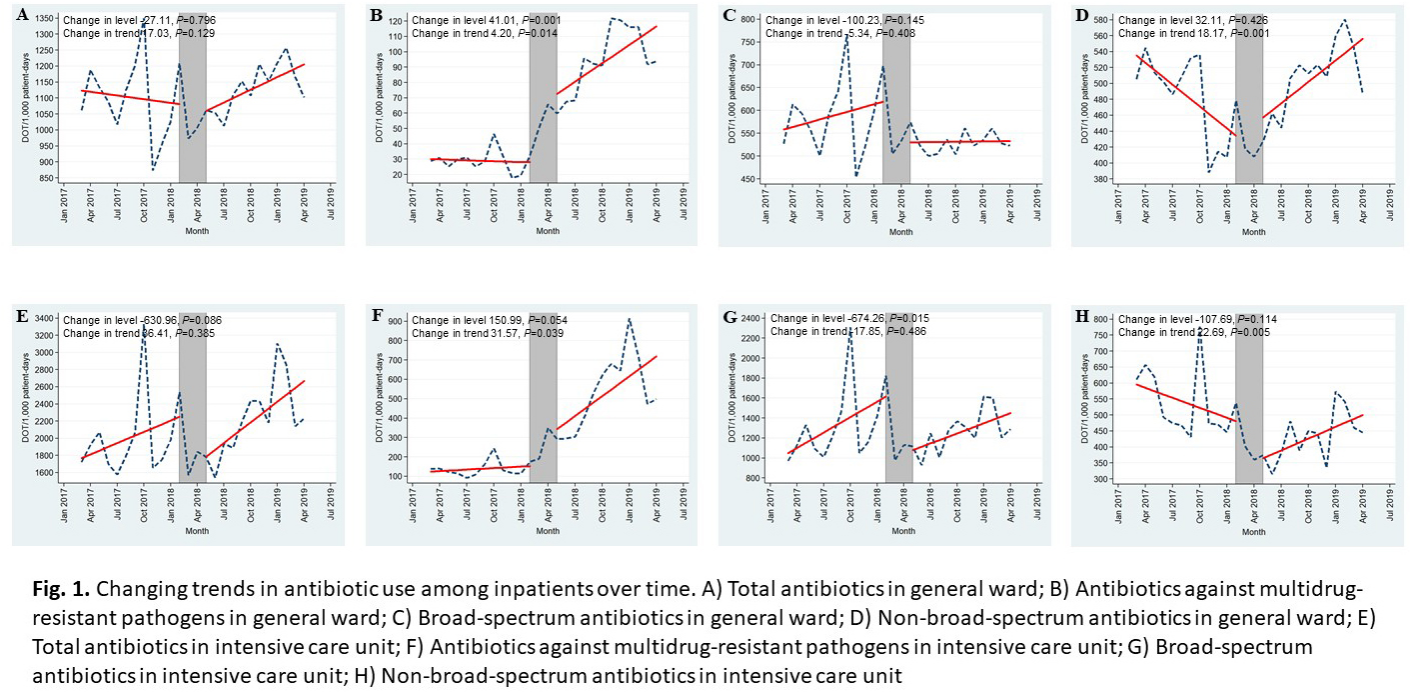

**Conclusion:**

In conclusion, after discontinuation of ASP, antibiotic usage patterns rapidly returned to the patterns prior to ASP implementation.

**Disclosures:**

**All Authors**: No reported disclosures

